# Choosing Optimal Cutoff Frequencies for Filtering Linear Acceleration and Angular Velocity Signals Associated with Head Impacts Measured by Instrumented Mouthguards

**DOI:** 10.1007/s10439-024-03466-4

**Published:** 2024-02-25

**Authors:** Ryan Gellner, Mark Begonia, Steve Rowson

**Affiliations:** Virginia Tech (Biomedical Engineering and Mechanics), Blacksburg, VA USA

**Keywords:** Instrumented mouthguard, Head impact, Filter, Six-degree-of-freedom (6DOF)

## Abstract

Head impact sensors worn in the mouth are popular because they couple directly to the teeth and provide six-degree-of-freedom head measurements. Mouthpiece signal filters have conventionally used cutoff frequencies lower than recommended practices (Society of Automotive Engineers, SAE J211-1) to eliminate extraneous noise when measuring with live subjects. However, there is little information about the effects of filter choice on the accuracy of signals measured by instrumented mouthpieces. Lack of standardization in head impact measurement device post-processing techniques can result in data that are not comparable across studies or device brands. This study sought optimal filter cutoff frequencies for six-degree-of-freedom measurements made at the teeth using instrumented mouthguards. We collected linear acceleration and angular velocity signals at the head center of gravity (CG) using laboratory-grade instrumentation. We also collected and filtered similar six-degree-of-freedom measurements from an instrumented mouthguard using 24 cutoff frequencies, from 25 to 600 Hz. We transformed the measurements to linear acceleration at the center of gravity of the head (CG) using all kinematic variables at the teeth, optimizing linear and angular mouthguard cutoff frequencies with one equation. We calculated the percent error in transformed peak resultant linear acceleration and minimized the mean and standard deviation in error. The optimal cutoff frequencies were 175 Hz for linear acceleration and 250 Hz for angular velocity. Rigid impacts (3–5 ms duration) had higher optimal cutoff frequencies (175 Hz linear acceleration, 275 Hz angular velocity) than padded impacts (10–12 ms duration; 100 Hz linear acceleration, 175 Hz angular velocity), and all impacts together (3–12 ms duration; 175 Hz linear acceleration, 250 Hz angular velocity). Instrumented mouthpiece manufacturers and researchers using these devices should consider these optimal filter cutoff frequencies to minimize measurement error. Sport-specific filter criteria for teeth-based sensors may be warranted to account for the difference in optimal cutoff frequency combination by impact duration.

## Introduction

Wearable head impact sensors provide an opportunity to collect potentially injurious head kinematics directly from living humans. Users ranging from laypeople to biomechanics researchers employ these devices, primarily in athlete populations [1–5]. Mouthguard- or retainer-style sensors have become increasingly popular because of their potential to couple directly to the upper dentition and, hence, the skull [6]. These sensors traditionally use three linear accelerometers and three angular rate sensors to generate six-degree-of-freedom (6DOF) motion information for the head. Popular sensors available on the market have been compared comprehensively in controlled laboratory environments and on-field [1, 2]. However, the sensors’ inherent signal-processing algorithms vary widely and are a source of error between reported measurements and truth. Instrumented mouthguards are subject to additional limitations and sources of noise when compared to laboratory-grade instrumentation: lower sampling rates, lower instrumentation bandwidths, imperfect coupling, imperfect fixation of the sensors within the instrument, extraneous impact from the mouth closing suddenly, and the inability to capture directly at the center of gravity. Filters used by instrumented mouthguard post-processing algorithms have traditionally used low cutoff frequencies to reduce noise (50–300 Hz for both angular and linear measures [2]), but this approach introduces the possibility of affecting the underlying impact response signal through excessive attenuation. Post-processing differences, especially filtering, make comparisons across devices and studies challenging and potentially misleading if ignored [7].

The present study replicates the methods presented in [8]. That study sought an optimal filter for head impacts measured by a 6DOF laboratory-grade instrumentation package mounted externally to the center of gravity (CG). The theoretical basis for kinematic ground truth was that transformed linear accelerations (transformation from the teeth to the CG) should match linear acceleration measurements taken directly at the CG. This theoretical basis relies on the assumption of the headform acting as a rigid body during impact. Because angular velocity, angular acceleration, and linear acceleration are all used when transforming the linear acceleration signals to another point on the head, filter cutoff frequencies can be simultaneously optimized for linear and angular kinematics against a ground truth. Deviations in measured values of the response signals can lead to erroneous computations of linear acceleration at the CG. We have reproduced those methods using an impact-monitoring mouthguard to determine the optimal filters for this device.

This study aimed to identify an optimal combination of linear acceleration and angular velocity cutoff frequencies for the measurements made at the teeth using instrumented mouthguards. We have done so by exploiting the physics of rigid body mechanics and minimizing the error in transformed linear accelerations. This approach inherently minimizes linear and angular error simultaneously through the transformation equation.

## Methods

A boil-and-bite mouthguard (Prevent Biometrics, Edina, MN; software version 2.0.14) was clamped to 3D-printed teeth in the mouth of a medium National Operating Committee on Standards for Athletic Equipment (NOCSAE) headform. The NOCSAE headform is used by the NOCSAE for testing and certifying athletic headgear, including helmets. It is a biofidelic headform developed with athletic equipment as its end use case. Originally developed in the 1970s, it is used to this day for athletic equipment testing [9]. It has been shown to produce similar kinematic results to the well-documented Hybrid-III anthropomorphic test device (ATD) head [10], while also enabling more realistic fit for sports helmets [11]. We chose the NOCSAE headform because it is commonly used to test athletic equipment, and instrumented mouthguards are often used in sports. This study used the medium male headform to represent an average user.

We conducted head impact tests on a pendulum impactor using two impactor faces (rigid and padded). The resulting impact tests spanned impact durations from on-field collegiate football and rugby environments [3, 12, 13]. We filtered data from the mouthguard with a range of cutoff frequency combinations for angular velocity and linear acceleration measurements. We computed error as the difference between peak values of mouthguard measurements transformed to the CG and peak values of headform measurements taken directly at the CG. We then found the cutoff frequency combination that minimized error, considering both mean and scatter error.

### Experimental Setup

**Fig. 1:**
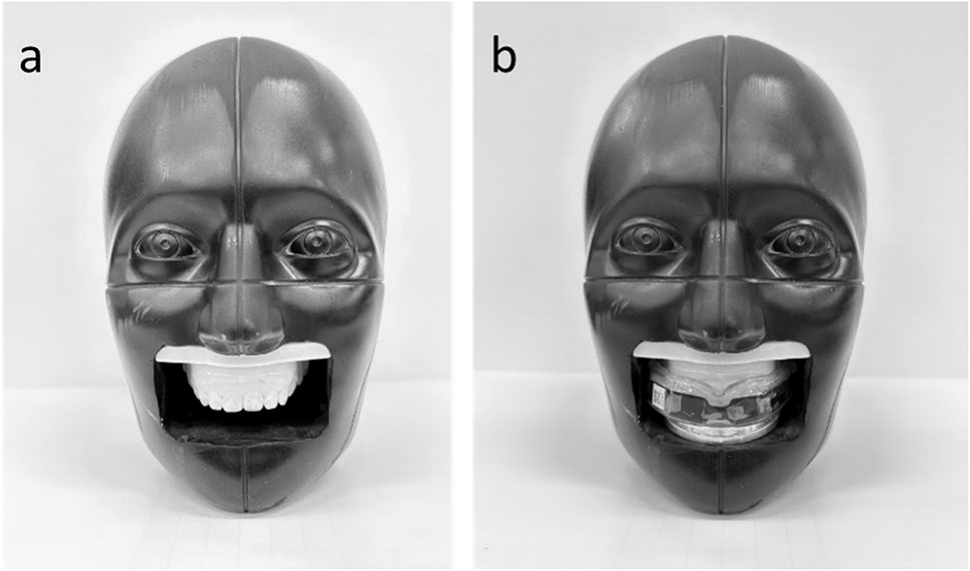
NOCSAE headform with **a** dental arches and **b** instrumented mouthguard clamped onto the dental arches via the aluminum plate below

The NOCSAE headform was modified so that 3D-printed dental arches could be mounted in an anatomically correct location for mouthguard testing (Fig. 1) [1]. Using the SAE J211-1 coordinate system, the mouthguard instrumentation was at + 82 mm along the *x*-axis, − 9 mm along the *y*-axis, and + 65 mm along the *z*-axis relative to the headform CG. We fit the boil-and-bite mouthguard to the teeth as the manufacturer’s instructions outlined. We removed the dental arches from the headform, boiled the mouthguard for 20 s, and then clamped the mouthguard to the dental arches for at least 60 s. We achieved the necessary clamping force via spring and trigger clamps (Pony Jorgensen). Finally, we confirmed the fit was acceptable by ensuring the mouthguard remained on the teeth under an open-mouth condition by pressing it onto the teeth and then letting go. The mouthguard did not fall off the teeth when no external pressure was applied. During testing, we tightened an aluminum plate onto the bottom surface of the mouthguard to clamp the mouthguard in place and represent a clenched-jaw condition.

Two impactor faces generated two linear acceleration durations. A rigid nylon face produced impacts of approximately 3–5 ms duration, while a padded face (VN600, Dertex Corporation) generated approximately 10–12 ms duration impacts. We estimated duration via visual inspection from the point at which the linear acceleration at the CG began to rise from the noise floor to the first local minimum with the greatest prominence. Estimates were a duration range because duration varied slightly across locations and target peak linear acceleration within each impactor face. Four locations were impacted on the headform (Rear, Rear boss, Front boss, and Front) using a pendulum at four different angles, as described in detail in previous studies [14]. These four pendulum angles were representative of target impact severities of 25 g, 50 g, 75 g, and 100 g. We accepted the test only if the impact resulted in a peak resultant linear acceleration at the CG of plus or minus 10% of the target linear acceleration. Any tests outside this range were repeated until the peak linear acceleration at the CG fell within this range. Each location, severity, and impactor face configuration underwent two trials for 64 total tests.

The mouthguard instrument included three linear accelerometers and three angular rate sensors, each sampling at 3.2 kHz. For the given sampling rate, the linear accelerometer had a bandwidth of 1600 Hz, while the gyroscope had a bandwidth of 890 Hz—both above the maximum cutoff frequency applied in this study (600 Hz). Signals measured from the mouthguard had 10 ms of pre-trigger and 40 ms of post-trigger samples. Mouthguard data collection was triggered when a single sample on any one linear accelerometer channel exceeded 8 g in the mouthguard sensor coordinate system*.* We instrumented the headform with three linear accelerometers (Endevco 7264b-2000) at the CG. Signals measured from laboratory devices included 50 ms of pre-trigger and 100 ms of post-trigger samples. Lab data collection was triggered when a single sample from the dominant axis exceeded 5 g (e.g., the *x*-axis for front impacts). The sampling rate of the lab instrumentation was 20 kHz, and the data acquisition system applied a hardware anti-aliasing filter with a cutoff frequency of 4 kHz to all channels before any other operations. We confirmed that the entire kinematic traces were contained within the data collection time window for all impacts in both systems.

### Theoretical Basis for Optimization Approach

We used a physics-based approach (Eq. 1) to generate optimal cutoff frequencies for linear and angular measures simultaneously. This approach relies on the assumption of the headform acting as a rigid body during impact. The equation for the transformation of linear acceleration included the variables of interest, which we could measure for both sides of the equation. When we use it to optimize filters, the transformation equation can be considered a function that requires angular velocity, angular acceleration, and linear acceleration measurements (right-hand side) to all align with truth (left-hand side). If any of these curves deviated from physical truth, the right-hand side would be in error. We considered the measurements taken from laboratory-grade instrumentation at the CG to be ground truth when filtered to SAE J211-1 specifications, though we acknowledge error is present in this measurement.

1 $$\overrightarrow {{a_{CG} }} = \overrightarrow {{a_{P} }} + \vec{\omega } \times \left( {\vec{\omega } \times \vec{r}} \right) + \vec{\alpha } \times \vec{r}$$

This approach is convenient in that we can optimize linear and angular measures at the same time. It also avoids the issue of ambiguity if one were to measure and compare angular velocity at two locations on an assumed rigid body: in this case, optimizing one angular measure to match another would not have a clear choice of ground truth, because all angular measures should theoretically be the same for a rigid body in motion. Thus, our approach provides a physics-based, unambiguous approach for optimizing linear and angular kinematic measurements.

### Data Processing

We began data post-processing with raw, unfiltered output signals from the mouthguard and laboratory instrumentation. The mouthpiece manufacturer did no post-processing on the mouthguard data before we received them. We removed bias by subtracting the average value of the first half of pre-trigger data from each signal. We removed bias before filtering for the mouthguard data to avoid errors caused by edge effects in the pre-trigger data when using extreme filter cutoffs. These edge effects were not present for the predetermined cutoff frequency used to filter lab data, so we removed bias after filtering the lab signals.

We filtered linear acceleration and angular velocity curves collected at the teeth using a 4th-order lowpass, phaseless digital Butterworth filter across a range of 24 cutoff frequencies (− 3 dB points) each, from 25 to 600 Hz in 25 Hz increments for a total of 576 filter combinations. The filter was equivalent to an SAE J211-1 filter and fell within the corridors for digital filters specified in that document [8]. Angular acceleration was calculated through five-point stencil differentiation of filtered angular velocity, as recommended in [15]. We filtered headform CG data to SAE-recommended practice: linear acceleration was filtered with a cutoff frequency of 1650 Hz, equivalent to CFC 1000. We then applied a rotation matrix to the CG and mouthguard signals to match SAE J211-1 coordinate axes definitions. The signals collected at the CG were offset by − 20° about the *Y*-axis, while mouthguard signals required matrix multiplication of the three axes by an orientation matrix (Appendix 1). Finally, we transformed linear acceleration from the teeth to the CG using Eq. 1 and the vector distance between the measurement locations described above (+ 82 mm, − 9 mm, + 65 mm).

Our method combined linear and angular measures to optimize cutoff frequencies for both. To verify that our method had properly optimized cutoff frequencies for angular and linear measures independent of one another, we included three angular rate sensors at the head CG (DTS ARS3 Pro 18k). Bias was removed for these sensors in the same way bias was removed from the linear accelerometers at the CG. The trigger, sampling rate, and hardware anti-aliasing filters were the same as the linear accelerometers at the head CG, as well. We filtered the ARS at the CG with a 300 Hz cutoff frequency, as prescribed by SAE J211-1. We applied the same rotation matrix to the ARS as was applied to the linear accelerometers so that they matched SAE J211-1 coordinate axes definitions.

We calculated the percent error by dividing the difference in peak resultant linear accelerations from the mouthguard by the peak resultant linear acceleration measured at the CG (Eq. 2). We then computed the mean and standard deviation of the percent error for each filter combination to quantify the mean (fixed) and variance (scatter) error. Because neither average error nor variance from a reference are desirable in a measurement device, we took the resultant of the mean and standard deviation (root sum of squares, RSS) as the minimization objective for determining optimal and acceptable minimum cutoff frequency combinations. This minimized both mean and scatter error. In our verification step, we quantified the percent error in peak angular velocity and peak angular acceleration using the same equation.

2 $$\in_{\% } = \frac{{{\text{PLA}}_{{\text{T}}} - {\text{PLA}}_{{{\text{CG}}}} }}{{{\text{PLA}}_{{{\text{CG}}}} }} \times 100\%$$

In Eq. 1, $\in_{\% }$ represents percent error, PLA_T_ represents peak linear acceleration from the transformed teeth measurement, and PLA_CG_ represents peak linear acceleration from the measurement at the CG.

The mouthguard sensor did not correctly collect some rigid impacts or missed them entirely in a few cases. This data loss could have been due to inherent limitations with the mouthguard instrumentation (e.g., sampling rate) or due to data rejection algorithms within the mouthguard software (version 2.0.14). Sometimes, the mouthguard missed the primary impact but collected a secondary impact (e.g., when the headform carrier slid into the spring stopper at the end of the track). To ensure these data artifacts did not influence the optimal filter for properly collected data, we excluded impacts wherein the mouthguard missed the primary impact (8 impacts, 12.5%).

We completed all data post-processing in MATLAB R2023 (MathWorks—Natwick, MA). We used RStudio 2023.06.0 (R Foundation for Statistical Computing, Vienna, Austria) to calculate errors and generate plots.

## Results

For laboratory instrumentation in all tests, peak linear acceleration at the center of gravity averaged 61.2 ± 29.2 g (mean ± standard deviation) and ranged from 20.4 to 107 g; peak angular velocity averaged 18.9 ± 12.9 rad/s and ranged 3.5 to 44.5 rad/s; and peak angular acceleration averaged 3526 ± 1902 rad/s/s and ranged 915 to 9592 rad/s/s. In the following sentences, we present mouthguard averages, standard deviations, and ranges using the optimal filter cutoff frequencies from the combined dataset of both rigid and padded impacts. Peak linear acceleration measured by the mouthguard averaged 62.6 ± 29.5 g (mean ± standard deviation) and ranged from 22.5 to 115 g. Peak angular velocity, measured by the mouthguard, averaged 18.3 ± 11.9 rad/s and ranged from 3.4 to 40.0 rad/s. Peak angular acceleration (derived from mouthguard angular velocity measurements) averaged 3549 ± 1856 rad/s/s and ranged from 1051 to 8990 rad/s/s.

**Fig. 2:**
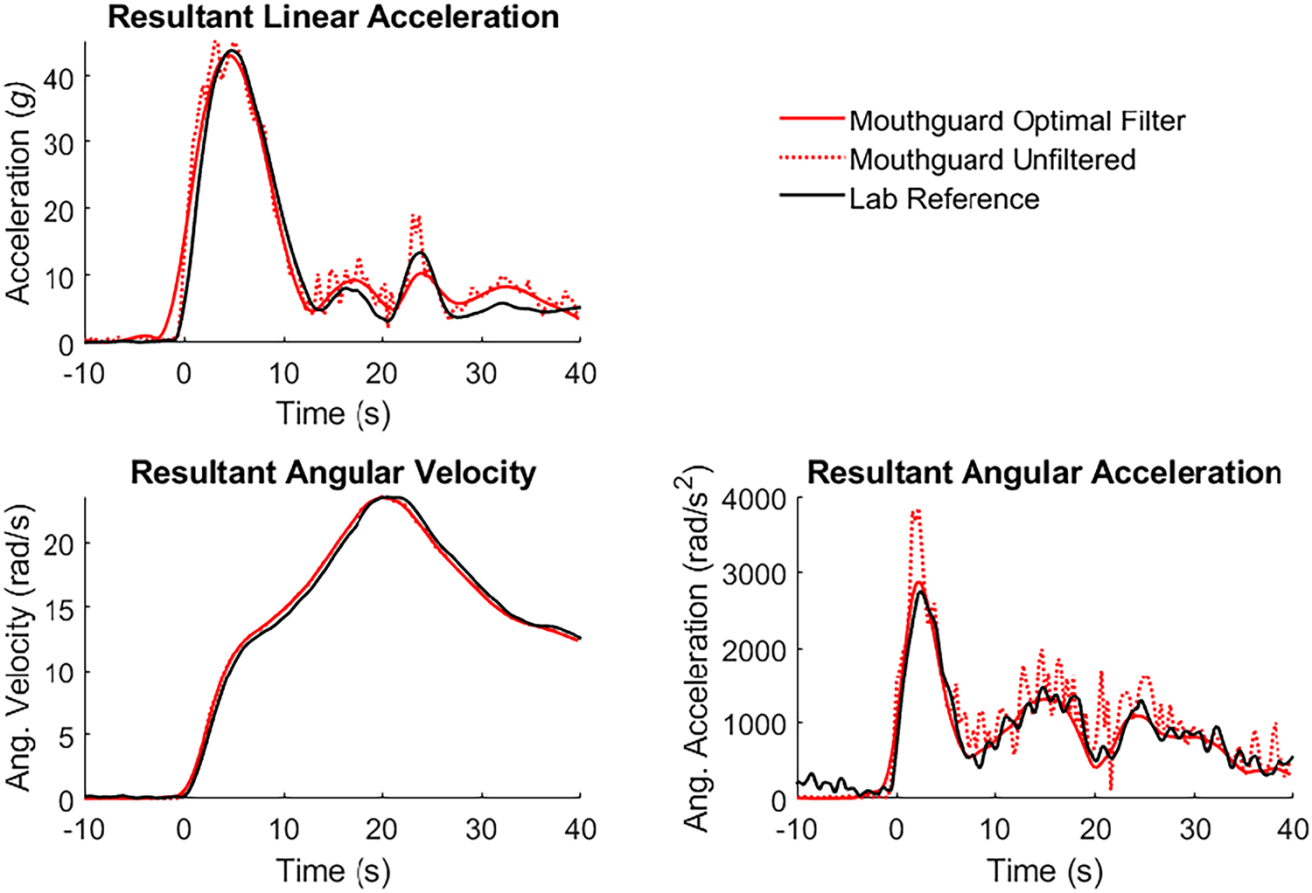
Exemplary resultant kinematic signal histories for a 50 g Front padded impact. Optimal filter signals used 100 Hz for linear acceleration and 175 Hz for angular velocity

The curves generally exhibited lower peak values with lower cutoff frequencies. Peak angular velocity was relatively insensitive to cutoff frequency because of its lower frequency content than acceleration signals. In contrast, linear and angular acceleration were more sensitive to cutoff frequency choice, because they contained higher-frequency content. The angular acceleration contains high-frequency noise from the differentiation step, while transformed linear acceleration contains high-frequency noise from the combination of linear acceleration, angular velocity, and angular acceleration. An example of the effects of the optimal cutoff frequencies on signals is shown in Fig. 2. The plot also demonstrates the error between the unfiltered signals and the lab reference measurement: unfiltered acceleration signals were visibly more oscillatory and had higher peaks than the reference signals.

When considering all impacts, the optimal cutoff frequency combination (i.e., lowest RSS error) for mouthguard measurements was 175 Hz for linear acceleration and 250 Hz for angular velocity. This combination had a mean error of 3.03%, a scatter error of 7.59%, and an RSS error of 8.17%. We also performed sub-analyses by impact duration, which was controlled by impactor face. Rigid impacts required a cutoff frequency combination similar to the combined dataset (175 Hz for linear acceleration and 275 Hz for angular velocity), which generated a mean error of 0.75%, a scatter error of 8.95%, and an RSS error of 8.98%. Padded impacts minimized error at a lower optimal cutoff frequency combination: 100 Hz for linear acceleration and 175 Hz for angular velocity. This optimal combination also generated a lower RSS error at 4.43% (mean error 1.15%, scatter error 4.28%) for these impacts. See Table 1 for notable cutoff frequency combinations.

**Table 1:** Percent error in transformed peak resultant linear acceleration

	Linear acceleration filter cutoff (Hz)	Angular velocity filter cutoff (Hz)	Mean % error (${\mu }_{e}$)	Std Dev % error (${\sigma }_{e}$)	RSS % ($\sqrt{{\mu }_{e}^{2}+{\sigma }_{e}^{2}}$)
Min. RSS—combined	175	250	3.03	7.59	8.17
Min. Mean—combined	100	350	− 0.02	11.6	11.6
Min. Std. Dev.—combined	200	300	7.27	7.02	10.1
CFC 180	300	300	11.2	9.09	14.4
Min. RSS—rigid impacts	175	275	0.75	8.95	8.98
Min. RSS—padded impacts	100	175	1.15	4.28	4.43

With all impacts, the cutoff frequency combination with the lowest absolute mean error was 100 Hz for linear acceleration and 350 Hz for angular velocity. This combination had a 0.02% mean error but an RSS error of 11.6% due to high variance. A wide range of cutoff frequency combinations had near-zero mean percent error. The cutoff frequency combination with the least scatter was 200 Hz for linear acceleration and 300 Hz for angular velocity. This combination had a mean error of 7.3%, a scatter error of 7.0%, and an RSS error of 10.1%. Linear and angular cutoff frequencies influenced mean error (Fig. 5, Appendix 2). Angular cutoff frequency influenced the scatter more than the linear cutoff frequency (Fig. 6, Appendix 2).

**Fig. 3:**
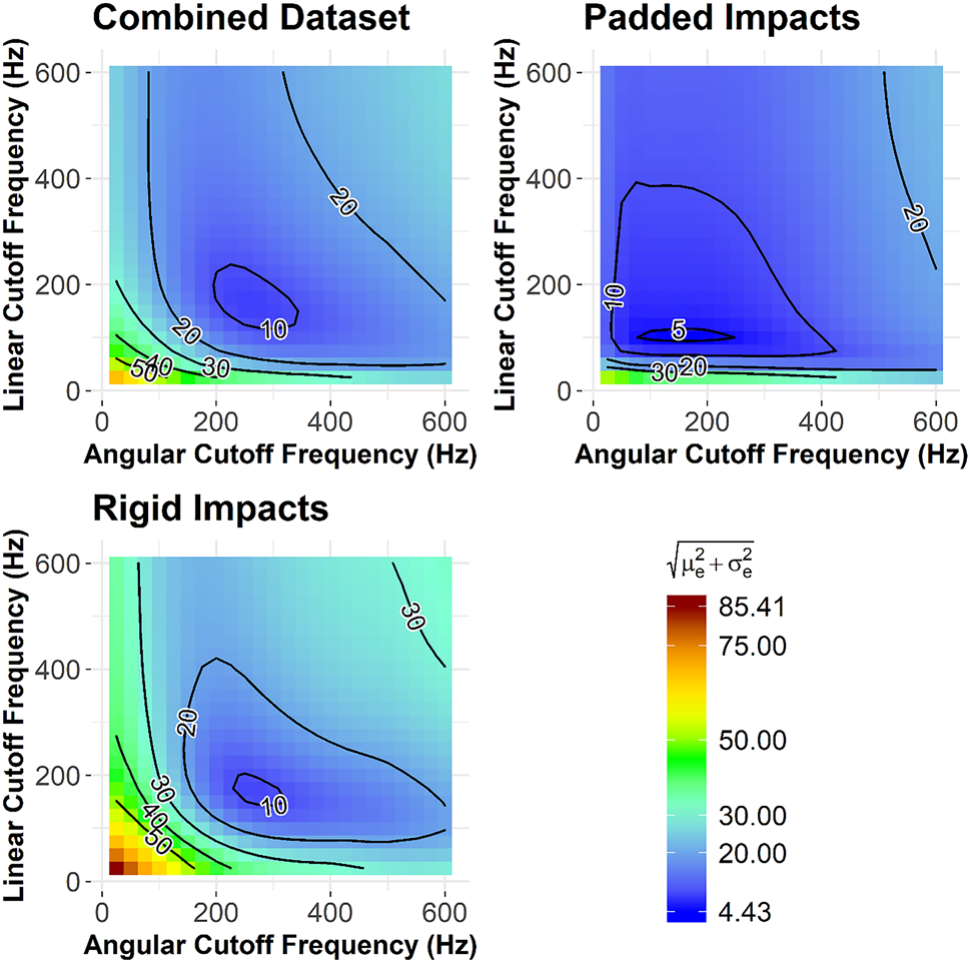
Resultant error contour plots by cutoff frequency are shown for the combined dataset (top left), longer-duration padded impacts (top right), and shorter-duration rigid impacts (bottom left)

The combined dataset had a region of cutoff frequency combination wherein impacts would have less than 10% RSS error on average (Fig. 3, top left). This region spanned an ellipse from approximately 125 to 250 Hz for linear acceleration and 200 to 350 Hz for angular velocity. We found a small area for padded impacts where the resultant error could be reduced to below 5%: angular velocity cutoff could range from 100 to 250 Hz, but linear acceleration cutoff had to remain at 100 Hz (Fig. 3, top right). We identified a 10% RSS error ellipse for rigid impacts from 150 to 200 Hz linear acceleration cutoff frequencies and 225 Hz to 325 Hz for angular velocity cutoff (Fig. 3, bottom left).

**Fig. 4:**
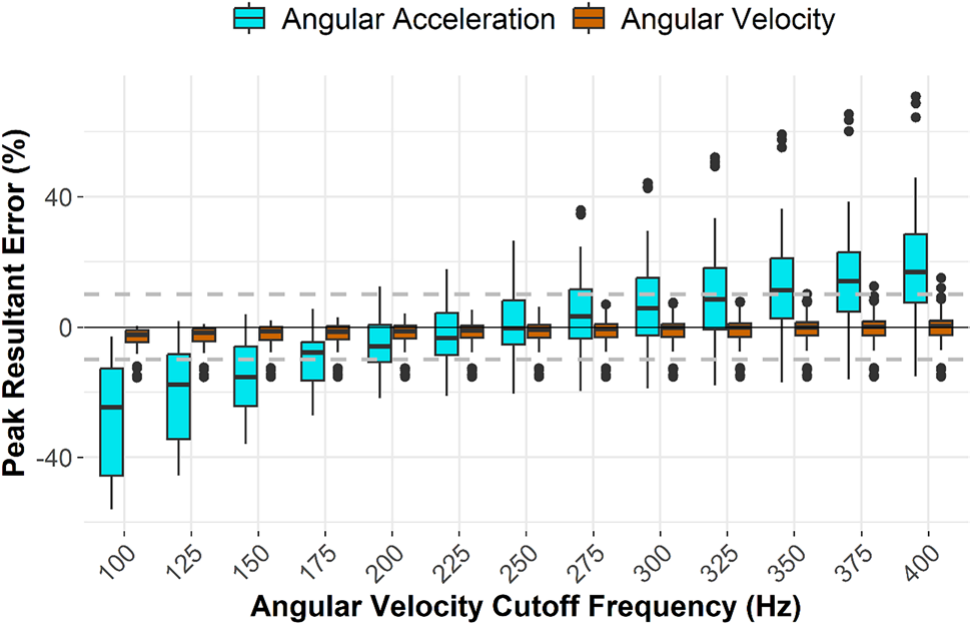
Distributions of the percent error in peak resultant angular kinematics from the mouthguard as a function of cutoff frequency applied to angular velocity curves. The black horizontal line represents 0% error, while the gray dotted lines are drawn at plus and minus 10% error

**Fig. 5:**
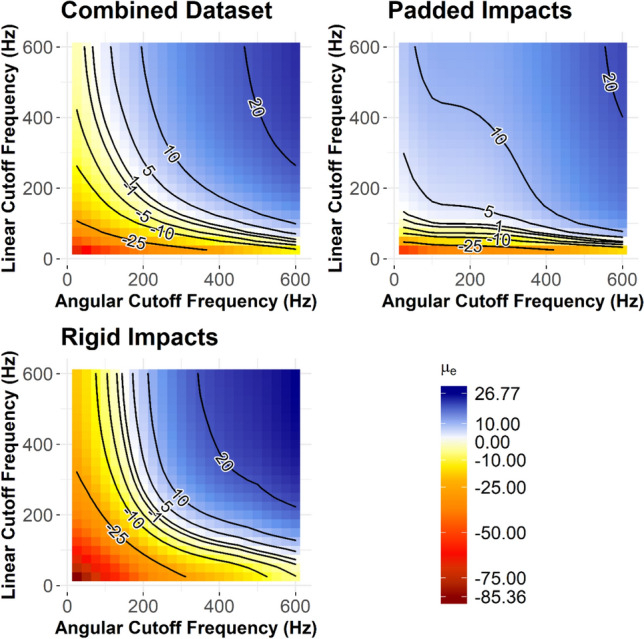
Mean error contour plots by cutoff frequency are shown for the combined dataset (top left), longer-duration padded impacts (top right), and shorter-duration rigid impacts (bottom left)

**Fig. 6:**
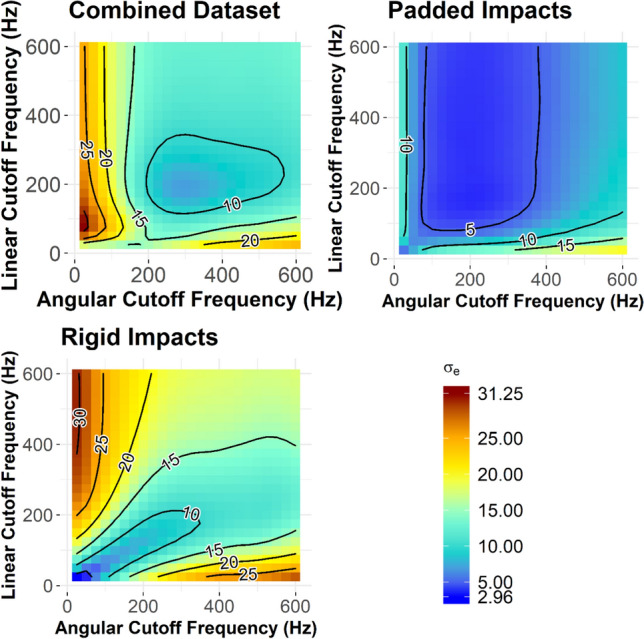
Standard deviation error contour plots by cutoff frequency are shown for the combined dataset (top left), longer-duration padded impacts (top right), and shorter-duration rigid impacts (bottom left)

In our verification step, we compared angular outputs from the mouthguard to those at the CG. Angular velocity and angular acceleration had slightly different error-minimization cutoff frequencies, with angular velocity preferring 300 Hz and angular acceleration preferring 250 Hz. However, the median angular velocity error was less than one percent across almost all tested cutoff frequencies. Therefore, the only cutoff frequency for the mouthguard that reduced median error in both angular velocity and angular acceleration to less than 1% was 250 Hz (Fig. 4).

## Discussion

### Optimal Cutoff Frequencies

Previous studies have compiled data from wearable sensors that filter transformed linear acceleration using various cutoff frequencies [2]. With instrumented mouthguards becoming prevalent and widely available, researchers can generate data from humans that will add to the more than 70 years of injury biomechanics literature. To do so effectively, data from these devices must be post-processed accurately. Researchers should avoid overestimating head kinematics due to cutoff frequencies that are too high and underestimating due to cutoff frequencies that are too low for the head’s impact response. Accurately estimating head kinematics is especially important as long as the head impact biomechanics community uses peak values to summarize impact severity.

In an idealized laboratory experiment with the mouthguard tightly coupled to the teeth, high-frequency noise can be introduced to the final transformed linear acceleration via the transformation process or a difference in frequency content occurring at the two locations. Both noise sources likely play a role [8]. Therefore, if cutoff frequencies that are too high are chosen for instrumented mouthguards, noise from both sources could leak into the final signals. Our data support this, showing that error increases past the optimal cutoff frequencies. This increase in error at high cutoff frequencies is similar to the increase in error at cutoff frequencies that are too low but not as exaggerated. Additionally, the error at higher cutoff frequencies is influenced more so by the angular cutoff frequency choice, indicated by vertical lines, whereas the error at low cutoff frequencies is influenced more so by the linear cutoff frequency choice, indicated by more horizontal lines. This error trend is similar to the trend identified in [8].

Transformed linear acceleration is calculated from three values in the present study: measured linear acceleration, measured angular velocity, and calculated angular acceleration. We used an SAE-recommended differentiation method to calculate angular acceleration from angular rate. However, differentiation still inherently amplifies high-frequency noise. In our study, the filters chosen for angular velocity are influenced by the error in angular acceleration. Thus, changing the differentiation technique or measuring angular acceleration directly may produce different optimal filters. The presented optimal filters are therefore valid for 6DOF (3a3w) systems, such as those commonly used in instrumented mouthguards today.

We verified our primary method by placing angular rate sensors at the headform CG. This allowed us to optimize angular measures independent of linear measures. The same cutoff frequency recommended for angular velocity in the full dataset (padded and rigid impacts) using the linear acceleration transformation method was also recommended for angular velocity using our secondary verification with angular measures only (250 Hz). This verifies that the transformation method minimizes error in both linear and angular measures, without over-filtering one to accommodate for the other.

### Comparisons to Previous Studies

The optimal cutoff frequencies identified in our study are consistent with the primary frequency content for each type of measured signal from the mouthguard: rigid impacts had primary frequencies below 150 Hz for both linear and angular mouthguard measurements, while padded impacts had primary frequencies below 50 Hz for both linear and angular measurements. We obtained these primary frequencies by selecting the frequency with the greatest amplitude in an FFT (MATLAB: *fft*) of the mouthguard signals per axis. The recommended optimal cutoff frequencies allow these primary frequencies to pass while removing unwanted frequency content. These primary frequencies are similar to those found by other authors using instrumented mouthguards in lab and field settings [6, 16–18].

The optimal cutoff frequency combinations presented here are slightly lower than those suggested in previous studies [8, 16, 17]. The lower optimal cutoff frequencies from the present study are likely because our study compared lower-grade wearable sensor signals to lab-grade instrumentation signals filtered at SAE J211-1 recommendations as ground truth. Previous studies have used full bandwidth signals [16], which means they were not filtered before finding the peak kinematics on the ground truth sensor. Our ground truth data were filtered, so we expect our optimal results to be lower. Our results are lower (100–275 Hz versus 300–500 Hz from Wu et al.), which aligns with our expectations. Cobb found an optimal angular velocity cutoff frequency of 292 Hz for laboratory instrumentation [17]. His results are very similar to the results from our previous study [8] of 300 Hz. Our previous study used the same methods as the current study but with laboratory instrumentation at the teeth instead of a commercial mouthguard. We expect our results here to be lower than those two studies, as well, because the mouthguard is less rigidly attached to the headform and has lower-grade instrumentation, a lower sampling rate, and a lower bandwidth. Our optimal cutoff frequencies are slightly lower than Cobb’s and Gellner’s, verifying our expectations.

Similar to those previous studies, rigid impacts were the limiting data because they have the highest frequency content. Lower cutoff frequency requirements are expected for longer-duration (padded) impacts because they inherently have lower frequency content. However, it is important to note that using the optimal cutoff frequency combination from the combined dataset (175 Hz linear, 250 Hz angular) for a dataset containing only padded (longer-duration) impacts would result in 6.6% ± 3.6% (mean ± standard deviation) error, an RSS error of 7.55%. This would result in 5.8 times higher mean error, only 17% lower scatter error, and 1.7 times higher RSS error than what is found using the optimal cutoff frequency combination for padded impacts alone.

### Practical Implications

Therefore, we suggest that sport-specific filter criteria for wearable, teeth-based sensors may be warranted. For sports with padded helmets and well-studied impact durations, American Football, for example [18], the longer-duration optimal filter for longer-duration impacts (100 Hz for linear acceleration, 175 Hz for angular velocity) is recommended for the lowest resultant error, minimizing both average and scatter error. However, for sports without helmets or where impact durations are unknown a priori, it is best to use the combined dataset's optimal cutoff frequency combination because an impact of any duration could be present in the data. If desired, these methods could be repeated for sport-specific impact durations to determine optimal filters for individual sports. If a mix of impact durations exists in a given sport, it may be useful to determine how frequently each duration type occurs and apply a weighted average to find an acceptable compromise between the optimal cutoff frequencies for the durations studied here. If future mouthguard technology enables reliable estimation of impact duration from individual signals, impact-specific cutoff frequencies may even be implemented; however, noise from field data in current technology may make this impractical for now.

The optimization was dominated by standard deviation error, but still identified target cutoff frequencies that generated low mean error. The final recommended cutoff frequency combinations have mean error less than or equal to 3%. Higher variance in rigid impacts led to greater RSS error, even with low mean errors. The lowest-error contour lines also covered less ground on the plots where rigid impacts were present. This implies that smaller changes in cutoff frequency can result in more considerable changes in resulting errors. Rigid, shorter-duration impacts would contain notably higher error than longer-duration impacts if not appropriately filtered because of their sensitivity to cutoff frequency. While these rigid impacts are relatively rare in most sports applications, they carry the highest risk of injury [19] and are therefore essential to report accurately.

The final recommended cutoff frequency combinations include some residual error, which can be mainly attributed to scatter error. The remaining error after optimization is inherent to the mouthguard sensors being less accurate than the laboratory-grade sensors, the differentiation process [15] adding high-frequency noise, and the breakdown in the assumption of rigidity in severe rigid impacts [8] causing different frequency content to be manifested at the two measurement locations. Reasonable error tolerance will ultimately be determined by the manufacturers and end users of these devices. Measurements should be both accurate and precise, and the RSS error reported in this study optimizes for both. The root sum of squares error combines average error with standard deviation error. RSS errors should not be compared to mean error alone or standard deviation error alone.

This study has limitations. We attempted to cover a range of impact durations (and thereby frequency content) commonly observed in head impacts from sports applications. However, some sports impacts may have different head impact durations outside this range. The optimal cutoff frequencies presented in this study may generate errors higher than those presented here if impact durations in practice are outside our tested range. The relative occurrence of padded and rigid impacts is not representative of most real-world applications of instrumented mouthguards. Rigid impacts are less common than what we have presented here; however, rigid impacts represent the upper bounds of cutoff frequency requirements and are relevant to this study as the limiting case. Our study experienced data loss from 8 rigid impacts due to limitations with the mouthguard. We don’t believe this meaningfully affected our results because the combined dataset was still most similar to the rigid results, implying that the rigid data were still well represented.

We used a single type of commercially available mouthguard to conduct this testing; however, the results fundamentally apply to the instrumentation grade, which is likely similar among commercially available head impact-monitoring mouthguards. Even still, other hardware may have slightly different optimal cutoff frequency combinations. This study applies to a 6DOF instrumentation package with three linear accelerometers and a triaxial angular rate sensor. Other instrumentation packages may have different optimal filters. We used the NOCSAE headform to conduct our testing. Past research has shown that this headform represents a human head in shape and size [10], and filter recommendations do not typically differ by headform [7]. Nonetheless, the use of a different headform may generate slightly different results. Finally, error was computed as the difference in peak resultant linear accelerations because peak values are sensitive to filter cutoff frequency, and head impact studies often report peak values to define impact severity [3, 20–22]. Other methods of determining error between signals may result in different optimal filter combinations.

In conclusion, we recommend that researchers and instrumented mouthpiece manufacturers use the optimal cutoff frequencies presented in this paper to minimize error in six-degree-of-freedom measurements. When head impact durations can be estimated with confidence based on previous research, we recommend using the duration-specific cutoff frequencies (i.e., padded or rigid) to minimize mean error and scatter.

## Appendix

### Appendix 1: Orientation Matrix for Mouthguard Data

We applied orientation matrices to assess mouthguard data in the SAE coordinate system. These orientation matrices are equivalent to three rotation matrices multiplied together.

For gyroscopes:

$$O = \begin{array}{*{20}c} {0.157} & { - 0.430} & { - 0.889} \\ { - 0.075} & { - 0.903} & {0.424} \\ { - 0.985} & {0.000} & { - 0.985} \\ \end{array}$$

For accelerometers:

$$O = \begin{array}{*{20}c} { - 0.903} & { - 0.424} & {0.174} \\ {0.430} & { - 0.889} & { - 0.075} \\ { - 0.157} & {0.000} & { - 0.985} \\ \end{array}$$

### Appendix 2: Percent Error Quantified by Mean and Standard Deviation

Both linear and angular cutoff frequencies influenced the mean error. Padded impact mean error was influenced more heavily by linear cutoff values (Fig. 5, top right). In contrast, rigid impacts displayed a strong influence from both cutoff frequencies (Fig. 5, bottom left). We observed rigid impacts, padded impacts, and the complete dataset (Fig. 5, top left) to have a narrow band traversing the plot in which filtered impacts had a near-zero mean error. Rigid impacts had generally higher magnitude percent error values than padded impacts. These findings are similar to previous studies using only laboratory instrumentation [8].

The minimum standard deviation of percent error in the combined dataset was 7.02% at 200 Hz linear acceleration cutoff frequency and 300 Hz angular cutoff (Fig. 6, top right). A large region kept the combined dataset impacts below 10% variance in error: from approximately 150 Hz to 300 Hz linear acceleration cutoff and about 200 Hz to 550 Hz angular velocity cutoff. Error variance changed more with angular velocity cutoff frequency than linear, which agrees with previous analyses [8]. Because frequency content is lower in longer-duration impacts, we found a larger region of low error variance for padded impacts (Fig. 6, top right) than for rigid impacts (Fig. 6, bottom left).
